# Cost-effectiveness of screening tools for identifying depression in early pregnancy: a decision tree model

**DOI:** 10.1186/s12913-022-08115-x

**Published:** 2022-06-13

**Authors:** Margaret Heslin, Huajie Jin, Kylee Trevillion, Xiaoxiao Ling, Selina Nath, Barbara Barrett, Jill Demilew, Elizabeth G. Ryan, Sheila O’Connor, Polly Sands, Jeannette Milgrom, Debra Bick, Nicky Stanley, Myra S. Hunter, Louise M. Howard, Sarah Byford

**Affiliations:** 1 Health Services and Population Research Department, Institute of Psychiatry, Psychology & Neuroscience at King’s College London, 16 De Crespigny Park, London, SE5 8AF UK; 2 Department of Statistical Science, University College London, London, UK; 3 Population, Policy and Practice Research & Teaching Department, UCL Great Ormond Street Institute of Child Health, London, UK; 4 King’s College Hospital NHS Foundation Trust, London, UK; 5 Centre for Health Services Research, University of Queensland, Brisbane, Australia; 6 Guy’s and St Thomas’ NHS Foundation Trust, London, UK; 7 Parent-Infant Research Institute, Austin Health, Heidelberg West, Victoria Australia; 8 Melbourne School of Psychological Sciences, University of Melbourne, Melbourne, Victoria Australia; 9 Warwick Clinical Trials Unit, University of Warwick & University Hospitals Coventry and Warwick NHS Foundation Trust, Coventry, UK; 10 School of Social Work, Care and Community, University of Central Lancashire, Preston, UK; 11 Department of Psychology, Institute of Psychiatry, Psychology & Neuroscience at King’s College London, Guy’s Campus, London, SE1 9RT UK

**Keywords:** Cost-effectiveness, Decision analytic model, Depression, Pregnancy, Screening, Whooley, EPDS

## Abstract

**Background:**

Although the effectiveness of screening tools for detecting depression in pregnancy has been investigated, there is limited evidence on the cost-effectiveness. This is vital in providing full information to decision makers. This study aimed to explore the cost-effectiveness of different screening tools to identify depression in early pregnancy compared to no screening.

**Methods:**

A decision tree was developed to model the identification and treatment pathways of depression from the first antenatal appointment to 3-months postpartum using the Whooley questions, the Edinburgh Postnatal Depression Scale (EPDS) and the Whooley questions followed by the EPDS, compared to no screening. The economic evaluation took an NHS and Personal Social Services perspective. Model parameters were taken from a combination of sources including a cross-sectional survey investigating the diagnostic accuracy of screening tools, and other published literature. Cost-effectiveness was assessed in terms of the incremental cost per quality adjusted life years (QALYs). Cost-effectiveness planes and cost-effectiveness acceptability curves were produced using a net-benefit approach based on Monte Carlo simulations of cost-outcome data.

**Results:**

In a 4-way comparison, the Whooley, EPDS and Whooley followed by the EPDS each had a similar probability of being cost-effective at around 30% for willingness to pay values from £20,000–30,000 per QALY compared to around 20% for the no screen option.

**Conclusions:**

All three screening approaches tested had a higher probability of being cost-effective than the no-screen option. In the absence of a clear cost-effectiveness advantage for any one of the three screening options, the choice between the screening approaches could be made on other grounds, such as clinical burden of the screening options. Limitations include data availability and short time horizon, thus further research is needed.

**Clinical trials registration:**

N/A

**Supplementary Information:**

The online version contains supplementary material available at 10.1186/s12913-022-08115-x.

## Background

### Context

Mental disorders are a significant problem during and after pregnancy for many women [1]. When experienced during pregnancy, mental disorders are associated with a variety of poor outcomes including low infant birth weight and preterm delivery [2–4], perinatal and infant death [5, 6], postnatal psychopathology [7–9], subsequent emotional and behavioural problems in the child and adolescent [10–13] and negative impact for other family members [14]. Depression is one of the most common mental disorders in pregnancy, with an estimated population prevalence in inner city maternity services of 11% [15]. Antenatal mental disorders, including depression, are often unrecognized and untreated [16], despite frequent contact with healthcare professionals throughout pregnancy. These contacts provide unique opportunities to identify and treat mental health problems in pregnant women.

### National guidance

The National Institute for Health and Care Excellence (NICE) [17] guidelines on antenatal and postnatal mental health recommends maternity professionals consider using the two Whooley questions [18, 19] to identify depressive disorders in pregnancy at the first antenatal appointment (8–10 weeks pregnancy) at which 86% of women are estimated to attend [20]. If a woman responds yes to either of the Whooley questions, the professional should consider referring the woman to her GP or mental health services. However, others advocate the use of the Edinburgh Postnatal Depression Scale (EPDS) [21].

### Existing evidence on the cost-effectiveness of screening for depression in the postnatal period

Hewitt and Gilbody [22] conducted a systematic review of economic evidence for screening for postnatal depression and found that there had been no studies on the cost-effectiveness in the area. Following this review being published, several studies have examined the cost-effectiveness of screening for depression in the perinatal period using economic models. Paulden et al. [23] examined the cost-effectiveness of routine screening for depression in primary care at 6 weeks postnatally via a decision model from an NHS and personal social services perspective over a 1-year time horizon. They compared routine clinical practice (no screening tool) with the EDPS and Beck Depression Inventory (BDI). They did not include the Whooley questions due to lack of data relevant to postnatal women available at the time. The authors reported that screening for postnatal depression was not cost-effective using the EPDS or BDIThe NICE guidelines [17] included a decision-analytic model from an NHS and personal social services over a 1-year time horizon to assess the relative cost effectiveness of identifying women with postnatal depression in the 6 weeks following childbirth. The guidelines compared the use of EPDS only, Whooley questions followed by the EPDS, and Whooley questions followed by the Patient Health Questionnaire-9 (PHQ-9) with routine clinical assessment (no screening tool). They concluded that the Whooley questions followed by PHQ-9 was the most cost-effective option. Wilkinson et al. [24] conducted a cost-effectiveness analysis of screening by physicians for postpartum depression and psychosis in the year following birth using a decision tree model with a 2-year time horizon from Medicaid payer perspective. They compared screening with the EPDS versus no screening (assuming that to have depression detected without screening, women had to choose to seek care for their depression). The authors reported that screening with the EPDS was cost-effective was around 85% at $27,500 (around the £20,000 NICE threshold). However, this incorporates the cost-effectiveness for screening for depression and psychosis combined.

### Existing evidence on the cost-effectiveness of screening for depression in the antenatal period

All of the above studies included the postnatal period only, therefore missing the opportunity to identify and respond to depression in pregnancy. Littlewood et al. [25] reported on the cost-effectiveness of screening for depression in the antenatal period within a decision model, from an NHS and social services perspective and a time horizon of 1 year after screening. They compared standard care case identification (no screening tool) with the following: the Whooley questions only; the EPDS only; the Whooley questions followed by the EPDS; and the Whooley questions followed by the PHQ-9. The authors reported that the Whooley questions followed by the PHQ-9 had the highest probability of being cost-effective with a probability of 0.47–0.48 for willingness to pay thresholds of £20,000–£30,000. This was followed by the Whooley questions followed by EPDS being the next most cost-effective option with a probability of 0.46–0.34 for willingness to pay thresholds of £20,000–£30,000. However, this study examined the cost-effectiveness of screening approaches at 20 weeks pregnancy (later than recommended by NICE), missing out on the opportunity to detect and treat depression early in pregnancy. There are a number of reasons why screening effectiveness and cost-effectiveness could be different if implemented at the first antenatal appointment compared to 20 weeks pregnancy resulting from emotional states relating to early pregnancy, anxiety in waiting for the first scan, and concerns about situation.

### Aim of this study

Therefore, the aim of this study was to explore the cost-effectiveness of the Whooley questions, the EPDS, and the Whooley questions followed by the EPDS, to identify antenatal depression compared to no screening tool at the first antenatal appointment.

## Methods

Although this study was conducted in conjunction with the cross-sectional survey conducted by Howard et al. [15] (described below), only a limited amount of the data were available from this work and much of the data is taken from elsewhere. The sources of data are described below.

This study was reported according to the CHEERS recommendations for reporting health economic evaluations [26].

### Target population and setting

The target population was pregnant women aged 16+ attending their first antenatal appointment with midwifes in South-East London, who do not have a miscarriage or termination between booking appointment and research interview. As described above, the first antenatal appointment was chosen because NICE recommends screening for depression in all pregnant women, and the first antenatal appointment is the first opportunity to screen the majority of women.

### Screening strategies

The following screening strategies were included:

- Whooley only - The Whooley questions are “During the past month, have you often been bothered by feeling down, depressed or hopeless?” and “During the past month, have you often been bothered by having little interest or pleasure in doing things?”. Answering yes to either question indicates a positive screen;
- EPDS only – The EPDS is a ten-item self-administered tool originally developed to assist in identifying possible symptoms of depression in the postnatal period. It also has adequate sensitivity and specificity to identify depressive symptoms in the antenatal period. A score of 13 or more was used to indicate a positive screen.
- Whooley followed by EPDS for those who are Whooley positive;
- No-screening (routine clinical assessment with midwives at the first antenatal appointment identifying depression via discussion and clinical judgement).

### Time horizon

From first antenatal appointment (approximately 8–10 weeks pregnant) to 36-week follow-up (3 months post-birth), a total of approximately 9-months.

### Model structure

**Fig. 1:**
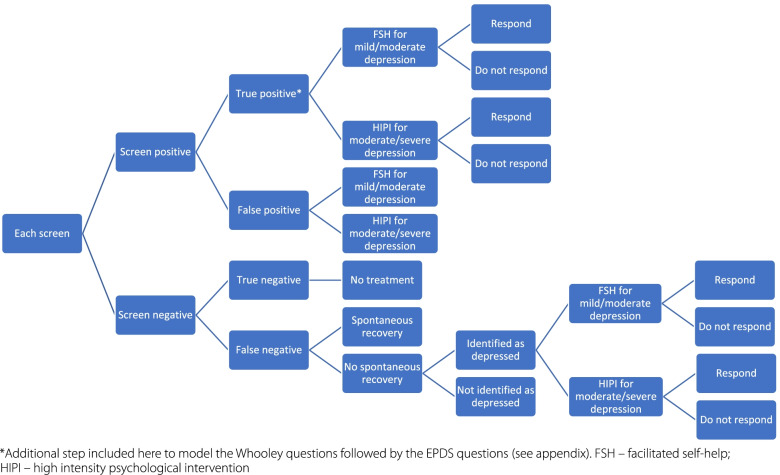
Detection and treatment model pathway

We developed a decision tree model in Microsoft Excel to evaluate the relative cost-effectiveness of the screening strategies. This model covered the pathway for detection and treatment (Fig. 1). At the start of the model, women receiving their first antenatal appointment are screened with either the Whooley, the EPDS, or the Whooley followed by the EPDS, or they receive no screen. Women who screen positive receive either facilitated self-help or high intensity psychological therapy depending on severity of symptoms from Improving Access to Psychological Therapies (IAPT) services, which they may, or may not, respond to. Women who screen negative receive no treatment. For those depressed women who are wrongly screened as negative (false negative), a proportion achieve spontaneous recovery. Of those who do not achieve spontaneous recovery, a proportion will be identified as depressed at a later point and receive treatment, whilst the remainder continue unidentified and receive no treatment for their depression. Model pathways were identical for all options except the Whooley followed by the EPDS, which required adaptation in order to model the two-stage screening process (see appendix). However, the treatment pathway was the same for all options.

### Model parameters

#### Clinical input parameters

Probabilities associated with the sensitivity and specificity of the screening tools, the treatment pathways modelled, response to treatment, and spontaneous recovery and later identification in false negatives are reported in Table 1. Data on sensitivity and specificity were taken from a cross-sectional survey conducted in a maternity service in South-East London which aimed to investigate the diagnostic accuracy of the Whooley questions and EPDS at the first antenatal appointment (see paper for full details) [15]. The Structured Clinical Interview DSM-IV (SCID) [27] was used as the ‘gold-standard’ diagnostic instrument to determine diagnosis and thus the accuracy of each screening approach. It is a semi-structured interview guide for making the mental health diagnoses and is administered by a clinician or trained mental health professional. Only the Axis I mood episodes, mood disorders and anxiety disorders module plus eating disorders, and SCID-II personality disorders subsection module for borderline personality disorders were used. Diagnosis of major depressive disorder included mild, moderate and severe depressive episode and mixed anxiety/depression.

**Table 1:** Model parameters for screening accuracy and treatment pathway

Parametre	Base-case probabilities	Raw data probabilities based on	95% CI	Source	Distribution	Notes
**SCREENING PATHWAY**						
***Whooley***						
Whooley positive	0.0909	906	0.085–0.097	Howard et al., 2018^a^ [15]	Beta	–
Whooley negative	0.9091	9057	0.903–0.915	Howard et al., 2018^a^ [15]	Beta	–
Whooley positive - true positive	0.4530	410.4	0.420–0.485	Howard et al., 2018^a^ [15]	Beta	–
Whooley positive - false positive	0.5470	495.6	0.515–0.580	Howard et al., 2018^a^ [15]	Beta	–
Whooley negative - true negative	0.9341	8460	0.929–0.939	Howard et al., 2018^a^ [15]	Beta	–
Whooley negative - false negative	0.0659	596.8	0.061–0.071	Howard et al., 2018^a^ [15]	Beta	–
***EPDS***						
EPDS positive	0.1144	1138	0.108–0.121	Howard et al., 2018^a^ [15]	Beta	–
EPDS negative	0.8856	8809	0.879–0.892	Howard et al., 2018^a^ [15]	Beta	–
EPDS positive - true positive	0.5188	590.6	0.490–0.548	Howard et al., 2018^a^ [15]	Beta	–
EPDS positive - false positive	0.4813	547.9	0.452–0.510	Howard et al., 2018^a^ [15]	Beta	–
EPDS negative - true negative	0.9534	8398	0.949–0.958	Howard et al., 2018^a^ [15]	Beta	–
EPDS negative - false negative	0.0466	410.3	0.042–0.051	Howard et al., 2018^a^ [15]	Beta	–
***Whooley-EPDS***						
Whooley positive	0.0895	890.2	0.084–0.095	Howard et al., 2018^a^ [15]	Beta	–
Whooley negative	0.9105	9057	0.905–0.916	Howard et al., 2018^a^ [15]	Beta	–
Whooley positive, EPDS positive	0.4114	366.2	0.379–0.444	Howard et al., 2018^a^ [15]	Beta	–
Whooley positive, EPDS negative	0.5886	524	0.556–0.621	Howard et al., 2018^a^ [15]	Beta	–
Whooley positive, EPDS positive - true positive	0.7500	8460	0.741–0.759	Howard et al., 2018^a^ [15]	Beta	–
Whooley positive, EPDS positive - false positive	0.2500	596.8	0.241–0.259	Howard et al., 2018^a^ [15]	Beta	–
Whooley positive, EPDS negative - true negative	0.7531	274.6	0.708–0.797	Howard et al., 2018^a^ [15]	Beta	–
Whooley positive, EPDS negative - false negative	0.2469	91.55	0.203–0.291	Howard et al., 2018^a^ [15]	Beta	–
Whooley negative - true negative	0.9341	394.6	0.910–0.953	Howard et al., 2018^a^ [15]	Beta	–
Whooley negative - false negative	0.0659	129.4	0.047–0.090	Howard et al., 2018^a^ [15]	Beta	–
***No-Screen***						
No-screen positive	0.0438	6	0.016–0.084	Hearn et al., 1998 [28]	Beta	–
No-screen negative	0.9562	131	0.916–0.984	Hearn et al., 1998 [28]	Beta	–
No-screen positive - true positive	0.6667	4	0.284–0.947	Hearn et al., 1998 [28]	Beta	–
No-screen positive - false positive	0.3333	2	0.053–0.716	Hearn et al., 1998 [28]	Beta	–
No-screen negative - true negative	0.8855	116	0.826–0.934	Hearn et al., 1998 [28]	Beta	–
No-screen negative - false negative	0.1145	15	0.066–0.174	Hearn et al., 1998 [28]	Beta	–
**TREATMENT PATHWAY**						
***Treatment***						
Facilitated self help for mild/moderate depression	0.7921	79.21	0.705–0.864	Howard et al., 2018 [15]	Beta	Assuming 50% of women with moderate depression receive this treatment
High intensity psychological therapy for moderate/severe depression	0.2079	20.79	0.136–0.295	Howard et al., 2018 [15]	Beta	Assuming 50% of women with moderate depression receive this treatment
***Spontaneous recovery***						
Spontaneous recovery	0.3300	33	0.242–0.425	Dennis et al., 2009 [29]	Beta	Midpoint of spontaneous recovery rate (25–40% = 33%).
No spontaneous recovery	0.6700	67	0.575–0.758	Dennis et al., 2009 [29]	Beta	One minus midpoint of spontaneous recovery rate.
***Later identification***						
Identified as depressed following first antenatal appointment	0.1025	10.25	0.050–0.166	Kessler et al., 2002 [30]	Beta	Based on 41% of misdiagnoses identified over the following 3 years.
Not identified as depressed following first antenatal appointment	0.8975	89.75	0.834–0.950	Kessler et al., 2002 [30]	Beta	One minus rate of identification.
***Response to treatment***						
Respond to facilitated self help	0.5109	51.09	0.413–0.607	NICE 2014 [17]	Beta	One minus probability of not responding.
No response to facilitated self help	0.4891	48.91	0.393–0.587	NICE 2014 [17]	Beta	Relative risk of no improvement (0.73) reported in NICE (2014) [17] multiplied by absolute risk of no improvement (0.67) reported by Dennis et al. (2009) [29] reported above.
Respond to high intensity psychological therapy	0.6784	67.84	0.586–0.767	NICE 2014 [17]	Beta	One minus probability of not responding.
No response to high intensity psychological therapy	0.3216	32.16	0.233–0.414	NICE 2014 [17]	Beta	Relative risk of no improvement (0.48) reported in NICE (2014) [17] multiplied by absolute risk of no improvement (0.67) reported by Dennis et al. (2009) [29] reported above.

^a^Data weighted to account for the bias induced by the stratified sampling

Data was not available from the cross-sectional study mentioned above [15] on the probabilities associated with the no screen alternative. Therefore, two rapid literature searches were conducted (1: in Ovid MEDLINE using keywords for perinatal, depression and screening; 2: in MEDLINE using keywords for perinatal, depression, midwifery, updated on 28th April 2021) and reference lists of relevant literature were searched to identify appropriate data. Although our model focuses on screening by midwifes at the pregnancy booking appointment, searches were widened to include the whole perinatal period and screening by any health professionals since we anticipanted very little data on screening in pregnancy by midwives. Additionally, we also considered data used by similar models regardless of the population. Four models and 4 studies with potentially relevant data were identified. Mitchell et al. [31] (used in models by Littlewood et al. [25] and NICE guideline [17]) presented data on the detection of depression by GPs. This was based on a systematic review and meta-analysis of GP depression diagnoses and reported a weighted sensitivity of 50.1% and weighted specificity of 81.3%. Kessler et al. [30] (used in the model by Paulden et al. [23]) estimated the probability that depression is missed at one routine primary care appointment and then detected 6 weeks later in routine primary care appointments. They reported that of 39% of people who had anxiety or depression and were assessed by their GP were identified as such by their GP. Both of these sources were considered to be inappropriate for the current model as the study focussed on depression in all people, not pregnant women. Wilkinson et al’s [24] paper on screening by physicians for postpartum depression and psychosis made the assumption that in the absence of a screening tool, women had to choose to seek care for their depression in order to receive treatment and estimated 34.2% of women with depression would seek help with no false positives. This was deemed to be inappropriate for our model as even before the introduction of screening tools in midwifery, midwifes would have a conversation about mental health with women to explore mental state.

Leverton et al. [32] presented data on health visitors ability to detect depression in the postnatal period. They reported a sensitivity of 8% and specificity 98%. Hearn et al. [28],presented data on midwives’ ability to detect mental health problems without a screening tool in the postnatal period. They reported a sensitivity of 21% and specificity 98%. As Hearn et al. [28] was based on data specifically from midwives, this was used to inform the model. However, Hearn et al. [28] used the EPDS to determine depression diagnosis rather than a clinical interview, and asked midwives to record “mental health problem” rather than depression. Therefore, this was varied in sensitivity analyses (described below).

In terms of treatment, we followed NICE guidelines (CG90 [33] and CG192 [17]). NICE [17] states that pregnant women with mild/moderate depression should be offered facilitated self-help (facilitated self-help) and pregnant women with moderate/severe depression should be offered a high-intensity psychological intervention. Since women with moderate depression can receive either facilitated self-help or high-intensity psychological intervention, we assumed 50% of women with moderate depression would receive facilitated self-help and 50% would receive high-intensity psychological intervention. We assumed that anyone who screened positive (whether a true positive or false positive) went on to have some treatment (see resource use section).

Data on the response to treatment was taken from a systematic review and meta-analysis (NICE guideline [17]). This reported the relative risk of no improvement following facilitated self-help and intensive psychological therapy in pregnant and postnatal women. The probability of response to facilitated self-help was calculated as 0.5109 (1-(absolute risk of no improvement multipled by probability of not responding following facilitated self-help); see Table 1; NICE [17]). The probability of response to high intensity psychological therapy was calculated as 0.6784 (1-(absolute risk of no improvement multipled by probability of not responding following high intensity psychological therapy); see Table 1; NICE [17]).

The probability of spontaneous recovery was taken from Dennis et al. [29] who discuss the fact that trials of treatment for postnatal depression report spontaneous recovery in controls groups of 25–40%. We applied the midpoint of 33%. This is consistent with the NICE guideline [17] estimate from meta analyses that the absolute risk of non-improvement is 67%, meaning spontaneous recovery rate is 33%.

To determine the probability of later identification in false negatives, literature was used from the rapid search on no screening alternatives described above. No study was identified that reported the probability of women with depression being detected following a negative screen. However, a study by Kessler [30] which reported on the probability that depression is missed at one routine primary care appointment and then detected later in routine primary care appointments was deemed to be a suitable alternative. The detection rate was reported as 41% over 3 years. Therefore, we adjusted this to 9-months and applied a 10% detection rate, assuming a linear relationship between time and detection, consistent with related models [17, 25].

#### Outcomes

Outcomes are described in Table 2. Utilities are preference weights which measure the health-related quality of life (HRQoL) of the individual at a particular point in time [34]. Utility is measured on a preference scale commonly anchored at 1 (perfect or best imaginable health) and 0 (death). Utility data for those with and without depression at the point of screening and at the end of the time horizon (3 months post-birth) were identified via a rapid search of the literature (run in MEDLINE using keywords for perinatal, depression, and quality of life, updated on 28th April 2021) and supplemented with hand searching of reference lists of related literature. Five papers were found with potentially relevant data. Four papers of these papers were not based on a perinatal population [35–38]. However, Littlewood et al. [25] reported utility data for ante-natal and postnatal depressed and non-depressed health states, based on the European Quality of Life-5 Dimensions-3 levels (EQ-5D-3 L [39]) from their cohort study. Since, these were the only perinatal utility values found, they were used in this model. Utility values were converted into QALYs using UK tariffs and taking the area under the curve approach by combining utility with time to create QALYs over the 9-months of the time horizon [40]. The QALYs are described in terms of depressed versus not in the ante-natal and post-natal period, ie, moving from depressed to non-depressed, starting depressed and remaining so, or starting non-depressed and remaining so.

**Table 2:** Model parameters for outcomes – utilities and QALYs

Parameter	Values	Source	Distribution	Standard error	95% CI
**Utilities**					
Ante-natal depressed	0.678	Littlewood et al., 2018 [25]	Beta	0.04	0.600–0.756
Ante-natal not depressed	0.888	Littlewood et al., 2018 [25]	Beta	0.01	0.868–0.908
Post-natal depressed	0.771	Littlewood et al., 2018 [25]	Beta	0.03	0.712–0.830
Post-natal not depressed	0.907	Littlewood et al., 2018 [25]	Beta	0.01	0.887–0.927
**QALYs (9 months)**					
Depressed to non-depressed	0.6553		Beta	30%	0.270–1.00
Depressed to depressed	0.5991		Beta	30%	0.247–0.951
Non-depressed to non-depressed	0.7422		Beta	30%	0.306–1.179

#### Resource use and unit costs

The economic evaluation took the NHS and Personal Social Services perspective preferred by NICE [41]. The costs associated with administering each screening approach, the costs of treatment and the costs of other health and social care costs are presented in Table 3. Data on the resources involved in screening and on other health and social care service use were identified through a rapid search of the literature (run in MEDLINE using keywords for perinatal, depression, screening and cost, updated on 20th April 2021) and supplemented with hand searching of reference lists of related literature. Only one study was identified that included data on resources involved in screening. These were taken from Littlewood et al. [25] Screening with the Whooley and EPDS were estimated to take 1.71 minutes and 3.54 minutes consecutively, and costs were attached to these from NHS reference costs [42]. The cost of the Whooley followed by the EPDS was calculated based on the costs for the Whooley and EPDS but with weighting for the proportion of people who need both screens (see Table 3). The cost of the no screen option was calculated as 3 minutes with a midwife (based on expert opinion that without a screening tool the midwife has a conversation about mental health of around 1–5 minutes).

**Table 3:** Model parameters for the cost of screening, treatment and other health and social care costs

Parameter	Cost (£)	Source	Distribution	Standard error	95% CI	Notes
**SCREENING**						
Whooley	4.53	Department of Health 2015/6 [42]	Gamma	Assumed to be 30% of the mean value	1.87–7.19	Based on 1.71 minutes to screen (Littlewood et al., 2018) [25] with a midwife costing £2.65 per minute (£53 per midwife appointment, average of 20 minutes per appointment, based on clinical opinion).
EPDS	9.38	Department of Health 2015/6 [42]	Gamma	Assumed to be 30% of the mean value	3.86–14.90	Based on 3.54 minutes to screen (Littlewood et al., 2018) [25] with a midwife costing £2.65 per minute (£53 per midwife appointment, average of 20 minutes per appointment, based on clinical opinion).
Whooley-EPDS	5.37	Department of Health 2015/6 [42]	Gamma	Assumed to be 30% of the mean value	2.21–8.53	Weighted cost based on the above – cost of Whooley screen for those who screen Whooley negative and cost of Whooley screen plus EPDS screen for those who screen Whooley positive, with proportions taken from the screening data in Table 1.
No-screening	7.95	Department of Health 2015/6 [42]	Gamma	Assumed to be 30% of the mean value	3.28–12.62	Based on 3 minutes with a midwife (expert opinion that without a screening tool the midwife has a conversation about mental health of around 1–5 minutes).^a^
**TREATMENT**						
Facilitated self-help	759	Radhakrishnan et al., 2013 [43]	Gamma	Assumed to be 30% of the mean value	312.71–1205.29	Based on seven face-to-face sessions (NICE, 2014) [17], at £98.59 per session (Radhakrishnan et al., 2013 [43]) based on 2009/10 prices, inflated to 2015/16 prices (Curtis and Burns, 2018 [44]).
High-intensity psychological intervention	3114	Radhakrishnan et al., 2013 [43]	Gamma	Assumed to be 30% of the mean value	1282.97–4945.03	Based on 16 sessions (NICE, 2014) [17], at £176.97 per session (Radhakrishnan et al., 2013 [43]) based on 2009/10 prices, inflated to 2015/16 prices (Curtis and Burns, 2018 [44]).
**OTHER HEALTH AND SOCIAL CARE**						
True positive who do not respond to treatment	2005	Petrou et al., 2002 [45]	Gamma	Assumed to be 30% of the mean value	826.06–3183.94	£2419 for women with depression over 18 months in 2000 prices inflated to 2015/16 prices (Curtis and Burns, 2018 [44]), and interpolated to 9 months.
True positive who respond to treatment	1680	Petrou et al., 2002 [45]	Gamma	Assumed to be 30% of the mean value	692.16–2667.84	£2027 for women without depression over 18 months in 2000 prices inflated to 2015/16 prices (Curtis and Burns, 2018 [44]), and interpolated to 9 months.
True negative	1680	Petrou et al., 2002 [45]	Gamma	Assumed to be 30% of the mean value	692.16–2667.84	£2027 for women without depression over 18 months in 2000 prices inflated to 2015/16 prices (Curtis and Burns, 2018 [44]), and interpolated to 9 months.
False negative	2005	Petrou et al., 2002 [45]	Gamma	Assumed to be 30% of the mean value	826.06–3183.94	£2419 for women with depression over 18 months in 2000 prices inflated to 2015/16 prices (Curtis and Burns, 2018 [44]), and interpolated to 9 months.
False positive	1680	Petrou et al., 2002 [45]	Gamma	Assumed to be 30% of the mean value	692.16–2667.84	£2027 for women without depression over 18 months in 2000 prices inflated to 2015/16 prices (Curtis and Burns, 2018 [44]), and interpolated to 9 months.

^a^Expert was a Consultant Midwife with over 40 years of clinical experience, and 20 years experience as a Consultant Midwife. She has held a variety of positions including as Midwifery Advisor to Department of Health 2005–2007, Midwife representative on Pan London Perinatal Psychiatry Clinical Network and NICE Guideline Development Groups for areas including, Social Exclusion, Caesarean Sections and Perinatal Mental Health. Her qualifications include MSc, BSc RM and RN

Data on other health and social care service use were required for those with and without a diagnosis at the point of screening and the end of the time horizon. Only one study was found to present health and social care service costs which could be used in the model: Petrou et al. [45] reported costs in mother-infant dyads over the first 18 months post-birth and reported costs by depressed and non-depressed women. This was inflated to the relevant year and applied.

Cost estimates for treatment were based on information obtained from the NICE guideline [17]. For true positives, the full treatment cost was assigned. For false positives, it was assumed they would receive the same treatments as true positives but that they would stop treatment earlier once their false positive status is recognised and would consume only 20% of treatment-related health-care resources, based on information reported in the NICE guideline [17]. It was assumed that women who screened negative would not receive any interventions after screening unless identified later.

Total costs for each arm are calculated by combining the cost of screening, treatment and other health and social care costs. All costs were in 2015/6 prices and reported in UK pounds sterling. Discounting was not used as the follow-up period did not exceed 12 months.

### Assumptions

The following assumptions were made, consistent with related models [17, 25]:

- All screening tools are used with all women at the first antenatal appointment;
- Women screened by antenatal services are not already receiving treatment for depression at the point of screening and therefore all women who screen positive will be referred for treatment;
- All women screened positive for depression are referred to IAPT, irrespective of the severity of depression’; All referrals to IAPT are accepted;
- No-one who screens negative and are true negatives at the first antenatal appointment become depressed following the appointment.

### Model outputs

Results are presented in three ways: average cost / average QALY gains per person; incremental cost-effectiveness ratios (ICERs); and cost-effectiveness planes and cost-effectiveness acceptability curves. ICERs are calculated by dividing the difference in total costs between two groups (incremental cost) by the difference in outcome between the two groups (incremental effect) to provide a ratio of ‘extra cost per extra unit of health effect’. Cost-effectiveness planes are used to visually represent the differences in costs and health outcomes between treatment alternatives (in this case screening alternatives), by plotting the costs against effects on a graph.

The analyses focused on the probability of each intervention being cost-effective compared with the others given the data available, which is the recommended approach for presenting evidence for decision-making, and is preferred over traditional reliance on arbitrary decision rules based on significance [46].

The mean cost and mean QALY gain per person are presented for each screening strategy. From this, the ICERs are calculated as the additional cost per QALY gain. When three or more alternatives are compared, ICERs are calculated using rules of dominance and extended dominance [47]. Cost-effectiveness planes and cost-effectiveness acceptability curves (CEACs) were produced using a net-benefit approach [48] based on Monte Carlo simulations of cost-outcome data from the probabilistic sensitivity analysis (described below). CEACs are an alternative to confidence intervals around ICERs and show the probability that one intervention is cost-effective compared to another, for a range of values that a decision maker would be willing to pay for an additional unit of outcome. They are graphs summarising the impact of uncertainty on the result of an economic evaluation. Four-way CEACs comparing all screening options simultaneously are presented.

#### Sensitivity analysis

The methods above describe the basecase analysis. The integrity of the results of economic models largely relies on the validity of the model input parameters and any assumptions made. Sensitivity analyses can be used to test the impact of changes in model parameters and assumptions on the results. If results from the sensitivity analyses are consistent with results from the base-case analysis, and would lead to similar conclusions about the cost-effectiveness of different strategies, one may be reassured that any uncertainty around the model input parameters and assumptions has little impact on the primary conclusions of the analysis. For this study, two types of sensitivity analyses were conducted: (1) deterministic sensitivity analyses to assess the impact of uncertainty around the value of individual parameters or uncertainty around the model structure and (2) probabilistic sensitivity analysis (PSA) to examine the impact of joint uncertainty of multiple parameters simultaneously. In a PSA, the uncertain parameters are characterised using probability distributions. Using Monte Carlo sampling methods, each model run draws a random sample from each uncertain parameter distribution. In the current study, this process was repeated 5000 times (bootstrap repetitions chosen a priori: see appendix for additional information on PSA convergence exercise), resulting in a joint distribution of cost and health outputs.

A range of one-way probabilistic sensitivity analyses were conducted:

- Detection in the no-screen pathway - The probabilities of the no-screen pathway were based on a study examining midwives’ ability to detect mental health problems without a screening tool [28]. However, this paper is from 1998 and reported very low rates of detection. Therefore, consistent with related models [17, 25], the probabilities associated with the no-screen pathway were replaced with those from a study on the detection of depression by GPs, and the cost of a GP contact replaced the cost of the nurse screening (as shown in Table 4) (sensitivity analysis 1a). Additionally, to challenge assumptions about costs and effectiveness of the no screening arm, we re-ran this analysis but replaced the cost of a GP contact with £0 (sensitivity analysis 1b).
- Treatment pathways – The basecase analysis assumed 50% of people with moderate depression would receive self-help and the other 50% would receive high-intensity psychological interventions. This was varied from 100% receiving self-help (sensitivity analysis 2a) to 100% receiving high-intensity psychological interventions (sensitivity analysis 2b).
- Later identification – The basecase analysis assumed that for false negatives, around 10% would be diagnosed later during the time horizon. This was adjusted to 5% (sensitivity analysis 3a) and 20% (sensitivity analysis 3b).
- Reduction in quality of life in false positives – The basecase analysis assumed that quality of life was not affected by being a false positive. However, this was adjusted to assume a 2% reduction in quality of life, in line with previous models (sensitivity analysis 4) [17].
- Utility for depressed and non-depressed states – Estimates of utility for depressed and non-depressed states came from published literature [25]. However, to test the impact of the utility values, we adjusted the utility for depressed groups by increasing (sensitivity analysis 5a) and decreasing (sensitivity analysis 5b) the utility for ante-natal and postnatal depressed states by 15%.
- Resource use by false positives – False positives were assumed to use 20% of the resources for treatment. This was adjusted to 10% (sensitivity analysis 6a) and 30% (sensitivity analysis 6b) in sensitivity analyses.
- Spontaneous recovery in the model was taken from a summary of studies reported by Dennis et al. [29] The methods of these studies somewhat limit the applicability here (including small sample sizes, based in different countries, post-partum rather than ante-natal populations and being dated). Therefore, we varied the spontaneous recovery rate to 0% (sensitivity analysis 7a) and 50% (sensitivity analysis 7b) in sensitivity analyses.

**Table 4:** Deterministic sensitivity analysis probabilities and cost parameters

Probabilities	Probability	Source	Data type	95% CI	Distribution	Notes
No-screen positive	0.2500	Mitchell et al., 2009 [31]	Binomial	0.171–0.339	Beta	
No-screen negative	0.7500	Mitchell et al., 2009 [31]	Binomial	0.661–0.829	Beta	
No-screen positive - true positive	0.4000	Mitchell et al., 2009 [31]	Binomial	0.221–0.594	Beta	
No-screen positive - false positive	0.6000	Mitchell et al., 2009 [31]	Binomial	0.406–0.779	Beta	
No-screen negative - true negative	0.8667	Mitchell et al., 2009 [31]	Binomial	0.782–0.933	Beta	
No-screen negative - false negative	0.1333	Mitchell et al., 2009 [31]	Binomial	0.067–0.218	Beta	
**Costs**	**Cost (£)**	**Source**	**Data type**	**95% CI**	**Standard error**	**Notes**
No-screen	31	Curtis & Burns, 2016 [49]	Assumed fixed	12.77–49.23	Assumed to be 30%	One GP appointment lasting 9.22 minutes, including direct care staff, no qualifications.

## Results

**Fig. 2:**
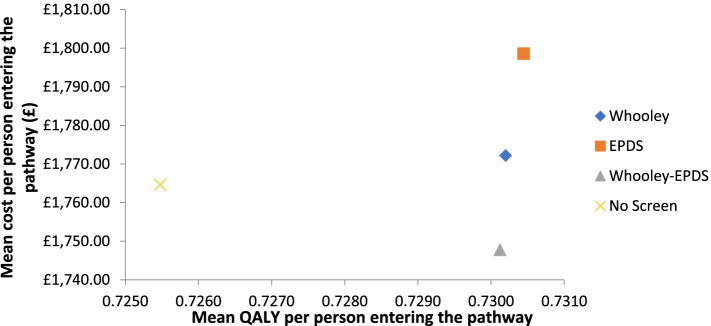
Costs and QALYs for each screening approach

The results of the basecase analysis are presented in Table 5 and Fig. 2. Mean QALY per person was highest for EPDS (0.7304), followed by Whooley (0.7302), Whooley-EPDS (0.7301) and no-screen (0.7255). Total cost per person was highest for EPDS (£1799), followed by Whooley (£1772), no-screen (£1765) and Whooley-EPDS (£1748). Using the rules of dominance and extended dominance, no-screen was dominated by Whooley-EPDS which was more effective and less costly. The incremental difference in QALYs per person compared to no screen was + 0.0049 for the EPDS, + 0.0047 for the Whooley, and + 0.0046 for the Whooley-EPDS. While the incremental difference in costs per person compared to no screen was +£34 for the EPDS, +£7 for the Whooley, and -£17 for the Whooley-EPDS. Hence the ICER for the EPDS, Whooley and Whooley-EPDS compared to no screen were £6939, £1489 and -£3696 per QALY respectively.

**Table 5:** Mean costs and QALYs for each screening approach

Screening approach	Mean QALYs	Mean Costs (£)	Incremental QALYs compared to no screen	Incremental costs compared to no screen	ICER compared to no screen
EPDS	0.7304	1799	0.0049	34	6939
Whooley	0.7302	1772	0.0047	7	1489
Whooley-EPDS	0.7301	1748	0.0046	−17	−3696

* No-screen dominated through rules of extended dominance so removed here (Mean QALYs: 0.7255; Mean costs: £1765)

A trade-off occurred for EPDS, Whooley and Whooley-EPDS, with EPDS costing more but producing more QALYs compared to the other strategies. Whooley-EPDS had the lowest cost of the remaining options but also produced the lowest QALYs. The ICER was £135,000 per QALY for EPDS versus the Whooley and £240,000 per QALY for Whooley versus Whooley-EPDS.

Results of the cost-effectiveness plane for Whooley versus EPDS, Whooley versus EPDS-Whooley and EPDS versus EPDS-Whooley all showed the scatter points were approximately equal in each of the four quadrants, suggesting no advantage for any option compared to the others in terms of costs or effects (see online appendix).

**Fig. 3:**
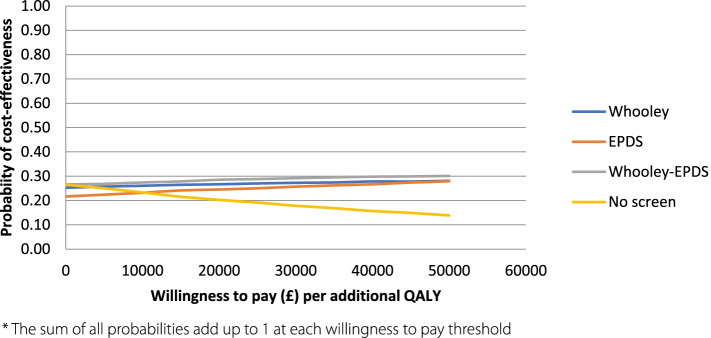
Cost-effectiveness acceptability curve for all screening approaches (basecase)

The cost-effectiveness acceptability curve (CEAC, Fig. 3) indicates that at a willingness to pay of £0 per QALY, all options have a similar probability of being cost-effective. However, as willingness to pay increases, the probability of no-screen being cost-effective falls, whilst the probability for all other screening options increase to a similar extent. At the £20,000–£30,000 cost per QALY threshold recommended by NICE, all three screening options have a higher probability of being cost-effective than the no-screen option.

**Fig. 4:**
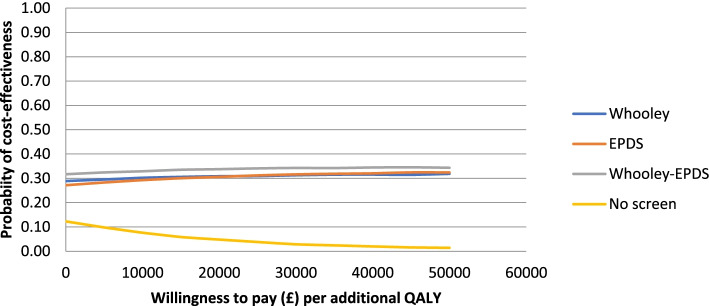
Sensitivity analysis 1a cost-effectiveness acceptability curve for all screening approaches (detection of depression using no screening tool adjusted)

The results of the sensitivity analysis 1a, where the detection of depression using no screening tool and the costs of no screening were adjusted using alternative sources of data, were similar to the basecase with no-screen being dominated, and the other screening options involving a trade-off. The 4-way CEAC (Fig. 4) confirms that at the £20,000–£30,000 cost per QALY threshold recommended by NICE, all three screening options have a higher probability of being cost-effective than the no-screen option. All other sensitivity analyses had similar results with each of the four screening approaches having a similar probability of being cost-effective at a willingness to pay of £0, but at the £20,000–£30,000 cost per QALY threshold, all three screening options have a higher probability of being cost-effective than the no-screen option (see online appendix).

## Discussion

### Main findings

This study compared three screening approaches against a ‘no screen’ alternative for detecting depression in pregnant women at their first antenatal appointment. In the base case analysis, the ‘no screen’ option was dominated by the other three options, with the Whooley, the EPDS and the Whooley followed by the EPDS all having a higher probability of being cost-effective than the no screen option at the £20,000–£30,000 cost per QALY threshold recommended by NICE. This was robust in sensitivity analyses where the probability of all four approaches being cost-effective was similar at very low levels willingness to pay amounts, but at the £20,000–£30,000 cost per QALY threshold, all three screening options have a higher probability of being cost-effective compared to the no screen option. The findings appear to be driven by the low cost of the screening interventions which all have similar sensitivity and specificity.

An apparent contradictory finding is that the Whooley followed by the EPDS has lower mean costs compared to the other options, even though the cost of a two-stage screening approach is higher than the alternatives. This is due to the fact that applying two screening tools sequentially increases the number of false negatives (participants falsely screened negative using the Whooley, who do not then proceed to the EPDS, plus further participants falsely screened negative using the EPDS) and fewer true positives (since more positives have been falsely screened negative). The impact is a reduction in the number of participants who are identified as true positive and proceed to treatment, compared to using one screening tool only. Since the cost of treatment is far higher than the cost of screening, this reduction in treatment costs (due to increased false negatives) far outweighs the increase in screening costs as a result of using a two-stage screening approach. Thus, the overall impact is to reduce the total cost of screening plus treatment.

Similarly, the Whooley followed by the EPDS had marginally lower mean QALYs compared to the Whooley alone and EPDS alone. This is also because the Whooley followed by the EPDS created more false negatives and less true positives leading to less opportunity to improve QALYs in people with depression, since a greater number of positive cases are falsely identified as negative and do not proceed to treatment.

The finding that combining two screening approaches leads to more false negatives and less true positives seems counter intuitive because one would assume combing tools would lead to better detection. However, by combining them we are simply create double the opportunity to incorrectly screen positive cases as negative for depression. Essentially the false negatives from both screen tools are combined.

The overall findings can be contrasted with those found by Littlewood et al. [25] who reported that the Whooley questions and the EPDS alone were never the most cost-effective strategy compared with the Whooley questions followed by the PHQ-9 and the Whooley questions followed by EPDS. Although the PHQ-9 was not part of this evaluation, the dominance of the Whooley followed by the EPDS in the Littlewood et al. study is at odds with the results presented in the current paper. This is likely to be a result of this study finding different levels of sensitivity and specificity for the Whooley, EPDS and the Whooley followed by the EPDS. These differences in sensitivity and specificity could be due to a number of differences between the studies including the use of midwives to ask the Whooley questions in the current study compared to researchers, differences in the population and study location (the current study included a more diverse population of women in inner-city London compared with a predominantly white, English-speaking population in a relatively rural area of the UK in Littlewood et al) [25], differences between the time points (8–10 weeks in this study versus 20 weeks in Littlewood et al. [25]), and use of the SCID as the gold standard in this study versus the CIS-R in Littlewood et al. [25]

### Strengths and limitations

This study included data from a cross-sectional survey specifically designed to compare the accuracy of alternative approaches to detecting depression in pregnant women at the first antenatal appointment. This is the earliest opportunity to systematically detect depression in pregnancy. Further, this study assessed the accuracy of the Whooley questions when asked by midwives at a routine maternity contact rather than validating responses to researchers, and thus the results are of relevance to usual clinical practice. Other strengths include the use of a robust diagnostic interview, an efficient, well-powered study design and a diverse study population.

A number of limitations which could have influenced the results should be considered. Although the Whooley questions were asked by midwives in clinical practice, the EPDS was administered by researchers. Therefore, the diagnostic accuracy of the EPDS may not reflect accuracy in clinical practice, although as it is a self-complete instrument its administration by researchers is unlikely to change its diagnostic accuracy. Further, there was a two to three-week delay in administering the EPDS and the SCID after the first antenatal appointment when the Whooley questions were asked so changes in mental state over this time period are possible. The model is also based on a number of key assumptions (e.g. all women are screened, no women are receiving IAPT prior to presentation, all who screen positive are referred to IAPT, and no-one who screened negative becomes depressed at a later point). However, assumptions are necessary in economic modelling as models are a simplification of reality. Further, these assumptions are consistent with related models [17, 25]. In relation to this, spontaneous recovery was simplified to allow analysis within the model. Spontaneous recovery was considered only in relation to false negatives and the impact of spontaneous recovery was not modelled in relation to true positives. Additionally, the resources, and therefore cost, of identification of depressed women in the no screening option were estimated based on the clinical opinion of a single Consultant Midwife with over 40 years of clinical experience, and 20 years experience as a Consultant Midwife. However, this estimate was varied in sensitivity analyses with no impact on the results.

The generalisability of the model must also be considered, as most data on the sensitivity and specificity of the screening tools came from one study based on one inner-city area, and screening data was only available for 33% of all eligible women. However, this is the first study to examine the cost-effectiveness of detecting and treating depression early in pregnancy informed by real world data on screening tool accuracy and there is flexibility in economic models to update the model parameters as additional data becomes available. Additionally, the time scale of this evaluation is limited to 3 months post-birth, thus any longer lasting impacts of detection and treatment of depression are not captured, and costs and benefits to the child are not considered.

Finally, at the time of this project starting, evidence of the effectiveness of the PHQ-9 was not available, therefore it was not included in this study. In light of previous work [25], the impact of using the PHQ-9 is likely to be important and thus is a limitation.

### Implications for policy

Since there was little difference in the cost-effectiveness of the three screening approaches tested and all were more likely to be cost effective at the £20,000–£30,000 cost per QALY threshold recommended by NICE, it would appear that any of the three alternatives are acceptable from an economic perspective and are preferred to a no-screen option. In the absence of a clear cost-effectiveness advantage for any one screening option, the decision could be made on other grounds, such as the clinical burden of the screening options. In this case, the ten questions of the EPDS could be potentially burdensome in busy maternity settings and it has been argued that the Whooley is the more favourable tool even in light of a slightly poorer diagnostic accuracy because of its brevity [15].

### Implications for further research

As with previous models in this area, we were unable to account for other mental health disorders as this was beyond the scope of this study. However, the impact of screening for depression and identification of other mental disorders, with associated referral and treatment pathways would impact the cost-effectiveness of screening approaches in a wider way. Additionally, the use of the PHQ-9 to detect depression once referral to IAPT has happened is important and should be examined if possible.

## Conclusions

The three screening approaches were more likely to be cost effective at the £20,000–£30,000 cost per QALY threshold recommended by NICE compared to the no screen option. In the absence of a clear cost-effectiveness advantage for any one of the three screening options, Whooley, EPDS, or Whooley and EPDS, the decision could be made on other grounds, such as clinical burden of the screening options. However, due to limitations of data availability and short time horizon, results should be viewed as provisional with the need for additional research.

## Supplementary Information

**Additional file 1.**

## Data Availability

The datasets generated during and/or analysed during the current study are not publicly available due to them containing sensitive identifiable information but are available from the corresponding author on reasonable request.
