# Supplementary material for: Cost-effectiveness of screening tools for identifying depression in early pregnancy: a decision tree model
Source: BMC Health Serv Res. 2022 Jun 13;22:774. doi: 10.1186/s12913-022-08115-x (PMC9190075; doi:10.1186/s12913-022-08115-x)
Supplement: Supplementary file 1 — Additional file 1. [file 12913_2022_8115_MOESM1_ESM.docx]

**Appendix 1**

**Figure: Detection and treatment model pathway for the Whooley questions followed by the EPDS questions screening approach**

**PSA convergence**

The number of PSA replications were chosen a priori so the replication number was independent of the results. This was prior to new guidance coming out on PSA convergence. New guidance recommends testing the stability of PSA results over different number of PSA runs (Hatswell et al. 2018). Therefore, PSA repetitions from 1,000-10,000 were run post-hoc. Results in the figure below indicated that stabilisation occurred around 3,000 replications, therefore 5,000 is sufficient.

Figure: PSA convergence

Reference:

Hatswell AJ, Bullement A, Briggs A, Paulden M, Stevenson MD. Probabilistic Sensitivity Analysis in Cost-Effectiveness Models: Determining Model Convergence in Cohort Models. Pharmacoeconomics. 2018 Dec;36(12):1421-1426. doi: 10.1007/s40273-018-0697-3. PMID: 30051268.

**Figure: Cost-effectiveness plane for Whooley versus EPDS (basecase analysis)**

**Figure: Cost-effectiveness plane for the Whooley compared to the Whooley followed by the EPDS (basecase analysis)**

**Figure: Cost-effectiveness plane for EPDS compared to the Whooley followed by the EPDS (basecase analysis)**

**Probabilities of cost-effectiveness for each approach for each sensitivity analysis at varying levels of willingness to pay**

|  |  | **Probability of being cost-effective at willingness to pay of:** | | | |
| --- | --- | --- | --- | --- | --- |
|  |  | **£0** | **£10k** | **£20k** | **£30k** |
| **Basecase** | Whooley | 0.25 | 0.26 | 0.27 | 0.27 |
|  | EPDS | 0.22 | 0.23 | 0.25 | 0.26 |
|  | Whooley-EPDS | 0.27 | 0.27 | 0.29 | 0.29 |
|  | No screening | 0.26 | 0.23 | 0.20 | 0.18 |
| **Sensitivity analysis 1a**  (Probabilities and costs of detection in no screen pathway adjusted) | Whooley | 0.29 | 0.30 | 0.31 | 0.31 |
|  | EPDS | 0.27 | 0.29 | 0.31 | 0.32 |
|  | Whooley-EPDS | 0.32 | 0.33 | 0.34 | 0.34 |
|  | No screening | 0.12 | 0.08 | 0.05 | 0.03 |
| **Sensitivity analysis 1b**  (Probabilities of detection in no screen pathway adjusted and cost adjusted to 0) | Whooley | 0.26 | 0.26 | 0.28 | 0.28 |
|  | EPDS | 0.22 | 0.23 | 0.25 | 0.26 |
|  | Whooley-EPDS | 0.27 | 0.27 | 0.28 | 0.29 |
|  | No screening | 0.26 | 0.23 | 0.20 | 0.17 |
| **Sensitivity analysis 2a**  (Treatment pathway adjustment from 50% of moderate cases receive self-help intervention in basecase to 100%) | Whooley | 0.25 | 0.26 | 0.27 | 0.28 |
|  | EPDS | 0.23 | 0.24 | 0.25 | 0.26 |
|  | Whooley-EPDS | 0.27 | 0.28 | 0.29 | 0.29 |
|  | No screening | 0.25 | 0.22 | 0.20 | 0.17 |
| **Sensitivity analysis 2b**  (Treatment pathway adjustment from 50% of moderate cases receive high-intensity intervention in basecase to 100%) | Whooley | 0.24 | 0.24 | 0.25 | 0.26 |
|  | EPDS | 0.23 | 0.24 | 0.25 | 0.26 |
|  | Whooley-EPDS | 0.28 | 0.29 | 0.30 | 0.31 |
|  | No screening | 0.25 | 0.22 | 0.19 | 0.17 |
| **Sensitivity analysis 3a**  (Percentage diagnosed later during the time horizon adjusted from 10% in basecase to 5%) | Whooley | 0.25 | 0.26 | 0.27 | 0.27 |
|  | EPDS | 0.22 | 0.23 | 0.24 | 0.25 |
|  | Whooley-EPDS | 0.27 | 0.28 | 0.28 | 0.29 |
|  | No screening | 0.26 | 0.23 | 0.21 | 0.19 |
| **Sensitivity analysis 3b**  (Percentage diagnosed later during the time horizon adjusted from 10% in basecase to 20%) | Whooley | 0.25 | 0.26 | 0.27 | 0.27 |
|  | EPDS | 0.22 | 0.24 | 0.25 | 0.25 |
|  | Whooley-EPDS | 0.27 | 0.28 | 0.29 | 0.29 |
|  | No screening | 0.26 | 0.22 | 0.20 | 0.20 |
| **Sensitivity analysis 4**  (Reduction in quality of life in false positives adjusted from 0% in basecase to 2%) | Whooley | 0.25 | 0.26 | 0.26 | 0.27 |
|  | EPDS | 0.22 | 0.24 | 0.25 | 0.26 |
|  | Whooley-EPDS | 0.27 | 0.28 | 0.29 | 0.30 |
|  | No screening | 0.26 | 0.23 | 0.20 | 0.18 |
| **Sensitivity analysis 5a**  (Utility for depressed and non-depressed states adjusted by increasing by 15% on basecase estimates) | Whooley | 0.25 | 0.25 | 0.25 | 0.26 |
|  | EPDS | 0.23 | 0.23 | 0.24 | 0.24 |
|  | Whooley-EPDS | 0.28 | 0.28 | 0.28 | 0.29 |
|  | No screening | 0.25 | 0.24 | 0.23 | 0.22 |
| **Sensitivity analysis 5b**  (Utility for depressed and non-depressed states adjusted by decreasing by 15% on basecase estimates) | Whooley | 0.26 | 0.28 | 0.30 | 0.30 |
|  | EPDS | 0.22 | 0.25 | 0.27 | 0.28 |
|  | Whooley-EPDS | 0.27 | 0.28 | 0.29 | 0.30 |
|  | No screening | 0.26 | 0.19 | 0.15 | 0.13 |
| **Sensitivity analysis 6a**  (Resource use by false positives decreased from 20% in the basecase to 10%) | Whooley | 0.24 | 0.26 | 0.27 | 0.27 |
|  | EPDS | 0.23 | 0.24 | 0.25 | 0.26 |
|  | Whooley-EPDS | 0.26 | 0.27 | 0.28 | 0.28 |
|  | No screening | 0.26 | 0.23 | 0.20 | 0.18 |
| **Sensitivity analysis 6b**  (Resource use by false positives increased from 20% in the basecase to 30%) | Whooley | 0.23 | 0.24 | 0.26 | 0.26 |
|  | EPDS | 0.22 | 0.23 | 0.24 | 0.24 |
|  | Whooley-EPDS | 0.28 | 0.29 | 0.30 | 0.30 |
|  | No screening | 0.27 | 0.24 | 0.20 | 0.20 |
| **Sensitivity analysis 7a**  (Spontaneous recovery rate reduced to 0%) | Whooley | 0.24 | 0.25 | 0.26 | 0.27 |
|  | EPDS | 0.23 | 0.25 | 0.26 | 0.27 |
|  | Whooley-EPDS | 0.26 | 0.27 | 0.28 | 0.29 |
|  | No screening | 0.26 | 0.23 | 0.20 | 0.17 |
| **Sensitivity analysis 7b**  (Spontaneous recovery rate increased to 50%) | Whooley | 0.25 | 0.25 | 0.26 | 0.26 |
|  | EPDS | 0.22 | 0.23 | 0.24 | 0.25 |
|  | Whooley-EPDS | 0.28 | 0.29 | 0.30 | 0.31 |
|  | No screening | 0.25 | 0.23 | 0.20 | 0.18 |

**Cost-effectiveness planes from sensitivity analyses**

Basecase cost-effectiveness plane

Sensitivity analysis 1 cost-effectiveness plane – alternative probabilities for the no screen alternative

Sensitivity analysis 2 cost-effectiveness planes – alternative treatment pathways

Sensitivity analysis 3 cost-effectiveness planes – alternative probabilities for later identification

Sensitivity analysis 4 cost-effectiveness plane – reduction in quality of life in false positives

Sensitivity analysis 5 cost-effectiveness planes – alternative utilities for depressed and non-depressed states

Sensitivity analysis 6 cost-effectiveness planes – alternative resource use by false positives

Sensitivity analysis 7 cost-effectiveness planes – alternative spontaneous recovery parameter
